# A Microbial Signature Identifies Advanced Fibrosis in Patients with Chronic Liver Disease Mainly Due to NAFLD

**DOI:** 10.1038/s41598-020-59535-w

**Published:** 2020-02-17

**Authors:** Tien S. Dong, William Katzka, Venu Lagishetty, Kayti Luu, Meg Hauer, Joseph Pisegna, Jonathan P. Jacobs

**Affiliations:** 1 The Vatche and Tamar Manoukian Division of Digestive Diseases, Department of Medicine, David Geffen School of Medicine at UCLA, Los Angeles, CA USA; 2 UCLA Microbiome Center, David Geffen School of Medicine at UCLA, Los Angeles, CA USA; 3 Division of Gastroenterology, Hepatology and Parenteral Nutrition, VA Greater Los Angeles Healthcare System, Los Angeles, CA USA

**Keywords:** Bacteria, Microbiota, Liver

## Abstract

The presence of advanced fibrosis is an important measure of the severity of chronic liver disease. Prior works that have examined the gut microbiome as a novel biomarker for advanced fibrosis have only examined patients with nonalcoholic fatty liver disease. Therefore, our goal was to examine the gut microbiome across varying etiologies of liver disease to create a predictive model for liver fibrosis based upon a microbial signature. Stool samples were obtained from patients with chronic liver disease (n = 50) undergoing FibroScan (ultrasound elastography) at the VA Greater Los Angeles Healthcare System. Healthy control patients (n = 25) were also recruited as a reference population. Fecal samples underwent 16S ribosomal RNA sequencing. Using differentially abundant microbes, a random forest classifier model was created to distinguish advanced fibrosis from mild/moderate fibrosis. The findings were then validated in a separate cohort of chronic liver disease patients (n = 37). Etiologies for liver disease included non-alcoholic liver disease (58.0%), hepatitis C (26.0%), hepatitis B (10.0%), and alcohol (6.0%). Microbiome composition was distinct in liver patients with advanced fibrosis compared to those with minimal fibrosis and healthy controls (p = 0.003). In multivariate negative binomial modeling, 26 bacterial taxa were differentially abundant in patients with advanced fibrosis as compared to those with minimal/moderate fibrosis (q-value < 0.05). A random forests classifier based on these taxa had an AUROC of 0.90 to predict advanced fibrosis. *Prevotella copri*, which was enriched in patients with advanced fibrosis, was the most strongly predictive microbe in the classifier. The classifier had an AUROC of 0.82 for advanced fibrosis in the validation cohort and *Prevotella copri* remained the strongest predictive microbe for advanced fibrosis. There is a distinct microbial signature for patients with advanced fibrosis independent of liver disease etiology and other comorbidities. These results suggest that microbial profiles can be used as a non-invasive marker for advanced fibrosis and support the hypothesis that microbes and their metabolites contribute to hepatic fibrosis.

## Introduction

Chronic liver disease is one of the most common medical conditions worldwide that affects as many as 840 million people with an estimated rate of mortality of 2 million deaths per year^1^. From 1999 to 2016, deaths from chronic liver disease in the US increased by 65 percent and deaths from liver cancer doubled^2^. This escalation has been attributed to factors such as increased alcohol use in younger Americans, increased intravenous drug use and rapidly rising rates of obesity in our society^2^.

Over the several years, there is a growing body of research investigating the role of the gut microbiome in disease pathology, and in particular, chronic liver disease^3^. The human microbiome has nearly 37 trillion cells and contains more than 100 times the number of genes than the human body^4^. While one of the principal function of the human microbiome is to ferment and create short-chain fatty acids from indigestible fiber, recent research studies have shown that the microbiome is also a key regulator of such liver diseases as NAFLD and primary sclerosing cholangitis^5–10^.

One of the most challenging aspects of chronic liver disease is the identification of patients with liver fibrosis. The development of advanced fibrosis is a major predictor of liver-related morbidity and mortality^11–13^. Early identification of advanced fibrosis using non-invasive testing is a growing area of research in the field of hepatology^12,14,15^. The characterization of gut microbial biomarkers for advanced fibrosis has been a novel area of ongoing research. For example, Qin and colleagues in 2014 reported that an intestinal microbial signature was present in individuals with cirrhosis in a Chinese cohort as compared to healthy controls^16^. This study included different causes of cirrhosis including hepatitis C, hepatitis B, NAFLD, and alcoholic liver disease. Loomba *et al*. in two separate studies was able to identify and validate a distinct microbial signature that was related to advanced fibrosis in patients with NAFLD^17,18^. However they did not explore other etiologies of chronic liver disease, so it is unclear at this time if this signature holds true for other causes of liver disease in western society. Given the association of the microbiome with chronic liver disease and cirrhosis, the aim of this study was to determine if specific fecal microbial profiles can be used as non-invasive biomarkers for advanced fibrosis in patients with varying etiologies of chronic liver disease.

## Methods

### Patient recruitment and stool collection

Patients with a diagnosis of chronic liver disease and undergoing ultrasound elastography were recruited prospectively from the VA Greater Los Angeles Healthcare System (VA) from 6/2017 to 6/2018. Chronic liver disease included patients with chronic hepatitis C virus (HCV) infection, chronic hepatitis B virus (HBV) infection, liver disease due to chronic alcohol use, primary biliary cholangitis (PBC), primary sclerosing cholangitis (PSC), Wilson’s disease, autoimmune hepatitis, hemochromatosis, and NAFLD. Patients were excluded if they were treated with antibiotics or probiotics within 3 months of enrollment, had only acute liver injury without any underlying chronic liver disease, treated HCV infection with sustained virologic response without any other forms of chronic liver disease, were on a specialized diet (e.g. gluten free, vegan, vegetarian, high protein), had a personal history of GI surgeries, irritable bowel syndrome or inflammatory bowel disease. Stool was collected within 7 days of their ultrasound elastography and placed into 95% ethanol and stored at −80°C until processing. Patient information including age, gender, race/ethnicity, and comorbidities were also collected. For race and ethnicity, there were 5 categories with Hispanic as a separate category (i.e. non-Hispanic white, non-Hispanic black, Hispanic, Asian, and other). Co-morbidities were collected in order to calculate the Charlson comorbidity index, a validated score that assesses overall health and risk of 1-year all-cause mortality^19^. Stool samples from heathy control patients without any evidence of chronic liver disease were also collected. The study was approved by the Veteran’s Affair Greater Los Angeles Healthcare System Institutional Review Board. All methods herein were performed in accordance with relevant guidelines and regulations. Verbal and written informed consent for study participation was obtained from all patients.

### Liver ultrasound elastography

All patients with chronic liver disease underwent an ultrasound elastography using the FibroScan touch 502 machine (Echosens, MA, USA). All ultrasound elastographies were performed by trained technicians with over 100 scans of experiences each. Medium (M) and extra-large (XL) probes were utilized depending on the patient’s body habitus according to manufacturer’s protocol. Controlled attenuation parameter (CAP) score and liver stiffness were collected as non-invasive measurements of hepatic steatosis and fibrosis, respectively. All measurements were done at least 10 times at the same spot with interquartile range/median value less than 30% as per manufacturers guidelines. A CAP score of between 238 and 260 was given a steatosis grade of S1 representing 11–33% of fatty change in the liver, a score between 260 and 290 was given a grade of S2 representing 34–66% of fatty change, and a score higher than 290 was given a grade of S3 representing 67% or more of fatty change as per manufacturer’s guideline. Standard cutoffs of liver stiffness as measured in kilopascals based on etiology of liver disease was used to determine extent of liver fibrosis (F0/F1 to F4)^20^. Minimal fibrosis was defined as a score consistent with F0-F2 and advanced fibrosis was defined as a score consistent with F3-F4, similar to prior published studies^17^.

### 16S rRNA sequencing

DNA was extracted from ethanol preserved stool using the Powersoil kit as per the manufacturer’s instructions (MO BIO, Carlsbad, CA, USA). The V4 region of 16S ribosomal RNA was amplified and underwent paired end sequencing on an Illumina HiSeq 2500 (San Diego, CA, USA) as previously described^21^. The 253 base-pair reads were processed using QIIME 1.9.1 (San Diego, CA, USA) with default parameters^22^. The average sequence depth per sample was 45,560. Operational taxonomic units (OTUs) were picked against the May 2013 version of the Greengenes database, prefiltered at 97% identity. After removing OTUs that were present in fewer than 10% of all samples, 1479 OTUs remained for analysis. Raw 16S rRNA sequence data were deposited under National Center for Biotechnology Information BioProject PRJNA542724.

### Statistical analysis

For demographic data, means are expressed along with their standard deviations and comparisons between means were performed using the Student’s t-test. Categorical data were compared using the Pearson’s chi-squared test.

For 16S rRNA sequencing data, alpha diversity metrics that included Chao1 (a metric for species richness), Faith’s phylogenetic diversity, and Shannon Index (a metric that incorporates both species richness and species evenness) were computed using QIIME. The statistical significance of differences in alpha diversity metrics was calculated using a two-tailed t-test. Beta diversity, a metric of differences between samples, was calculated using the square root of the Jensen-Shannon divergence and visualized by principal coordinates analysis in R^23^. Univariate Adonis, a permutational analysis of variance, was performed using 10,000 permutations to test for differences in the square root of the Jensens-Shannon divergence across the following variables: age, gender, race/ethnicity, BMI, control/patient cohort, fibrosis as a binary categorical variable, steatosis grade, etiology of liver disease, and Charlson’s comorbidity index. Only variables with a p-value < 0.1 were used for the final multivariate analysis. This included steatosis grade, Charlson’s comorbidity index, and fibrosis. Differential abundance testing was evaluated using DESeq2 in R, which employs an empirical Bayesian approach to shrink dispersion and fit non-rarified count data to a negative binomial model^24^. Variables listed in the multivariate analysis of DESeq2 were the same variables listed above for the multivariate Adonis analysis. P-values for differential abundance were converted to q-values to correct for multiple hypothesis testing (<0.05 for significance). All authors had access to the study data and had reviewed and approved the final manuscript.

### Random forests classifier

A random forests classifier to predict advanced fibrosis was created in R using the randomForest package (https://cran.r-project.org/web/packages/randomForest) with 1001 trees and mtry = 2^25^. Features inputted into the random forest classifier were those associated significantly with advanced fibrosis as determined by multivariate DESeq2 models. The accuracy of the random forest classifier was estimated using a 10-fold cross-validation.

### Predicted metagenomics

Metagenomic data of each sample was inferred from 16S rRNA sequencing data by using PICRUSt 1.1.3 (http://picrust.github.io/picrust), a well validated tool designed to impute metagenomic data from 16S rRNA compositional data^26^. 16S rRNA sequencing data was inputted into PICRUSt and normalized by copy number using default parameters. The subsequent metagenes were then categorized by function using the KEGG database. Differences in predicted metagenes by advanced fibrosis were identified using DESeq2 with p-values adjusted for multiple hypothesis testing.

### Validation cohort

The findings of the random forest classifier were validated in a separate cohort of NAFLD patients recruited at the VA from January 1^st^, 2019 to October 1^st^, 2019. Inclusion and exclusion criteria were the same as above. All patients underwent stool collection and liver ultrasound elastography as described above. Demographic data, race, ethnicity, and comorbidities were collected. In addition, all patients within this cohort filled out a validated diet questionnaire, the NIH Diet History Questionnaire III (DHQIII), at the time of their stool collection^27^.

### Synopsis

This is one of the few studies that have examined the microbiome as a novel biomarker for advanced fibrosis. Unlike prior works that have only examined patients with nonalcoholic fatty liver disease, this study included patients from various races and etiologies of liver disease. The study highlights how the gut microbiome may play a role in fibrosis progression.

## Results

### Patient and healthy control characteristics

Fifty patients with chronic liver disease and 25 healthy controls were recruited. Etiologies for liver disease included non-alcoholic liver disease (58.0%), hepatitis C (26.0%), hepatitis B (10.0%), and alcohol (6.0%) (Table 1). Nineteen patients had advanced fibrosis and 7/19 (36.8%) had F4 fibrosis. The healthy control cohort were younger on average than the patients with chronic liver disease and comprised of more females (p-value < 0.001). The average Charlson’s Comorbidity Index for the liver disease cohort was 4.33 ± 2.31. There was no difference in Charlson’s Comorbidity Index between patients with advanced fibrosis as compared to those without advanced fibrosis. There was no difference in race/ethnicity between any groups and there was no statistical difference in etiologies of chronic liver disease by fibrosis stage.

**Table 1:** Patient and Healthy Control Characteristics.

	Control (n = 25)	F0-F2 (n = 31)	F3/F4 (n = 19)	p-value
Age (yr) (SD)	35.7 (3.5)	58.7 (16.3)	66.2 (6.8)	**<0.001**
Male (%) (n = 60)	52% (n = 13)	88.9% (n = 28)	100% (n = 19)	**<0.001**
Charlson Comorbidity Index (SD)	N/A	3.9 (2.6)	5.1 (1.5)	0.13
Race/Ethnicity				
Caucasian (%) (n = 26)	32.0% (n = 8)	38.7% (n = 12)	31.6% (n = 6)	0.89
African American (%) (n = 26)	32.0% (n = 8)	32.3% (n = 10)	42.1% (n = 8)	
Hispanic (%) (n = 10)	8.0% (n = 2)	16.1% (n = 5)	15.8% (n = 3)	
Asian (%) (n = 7)	16.0% (n = 4)	6.5% (n = 2)	5.3% (n = 1)	
Other/Unknown (%) (n = 6)	12.0% (n = 3)	6.5% (n = 2)	5.3% (n = 1)	
Etiology of Liver Disease				
EtOH (n = 3)	N/A	6.5% (n = 2)	5.3% (n = 1)	0.18
HBV (n = 5)		12.9% (n = 4)	5.3% (n = 1)	
HCV (n = 13)		16.1% (n = 5)	42.1% (n = 8)	
NAFLD (n = 29)		64.5% (n = 20)	47.4% (n = 9)	

Fibrosis stage labeled from F0-F4. Minimal/no fibrosis: F0-F2; Advanced fibrosis: F3/F4; SD: standard deviation; EtOH: Alcohol, HBV: Hepatitis B virus, HCV: Hepatitis C virus, NAFLD: nonalcoholic fatty liver disease.

### Microbial profiles differs by fibrosis stage and etiology of liver disease

**Figure 1:**
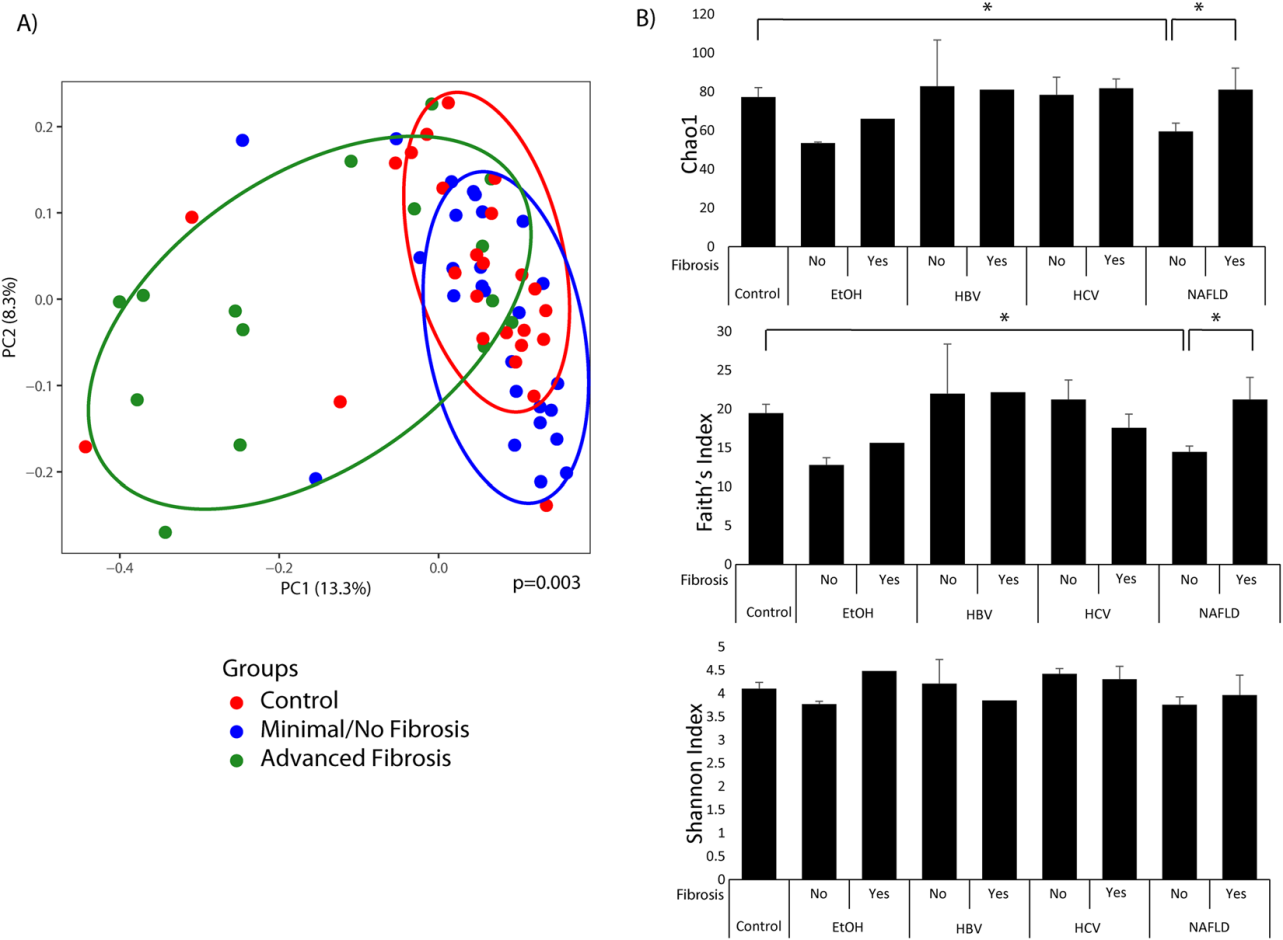
Patients with Advanced Fibrosis have Distinct Microbial Composition and Diversity Compared to Other Liver Disease Patients or Healthy Controls. (**A**) Beta diversity visualized by principal coordinates analysis plot of all patients colored by fibrosis stage or control group. (**B**) Alpha diversity metrics by etiology of chronic liver disease and fibrosis stage. Chao1 is a metric of species richness, Faith’s Index is a metric of phylogenetic diversity, and Shannon index is a metric of species richness/evenness. *Represents comparison with p-value < 0.05.

In univariate analysis of beta diversity, only 3 variables had a p-value < 0.1: steatosis grade, Charlson’s comorbidity index, and the presence of advanced fibrosis. Therefore, these variables were used for the multivariate analysis. As demonstrated in the principal coordinates analysis plot (Fig. 1A), the microbial profile of patients with advanced fibrosis differed significantly as compared to those with minimal or no fibrosis or healthy controls (p = 0.003), while adjusting for the other covariates. In regards to alpha diversity metrics, patients with NAFLD and minimal or no fibrosis had a lower Chao1 index (species richness) and a lower Faith’s Phylogenetic Diversity as compared to healthy controls and NAFLD patients with advanced fibrosis (Fig. 1B). There was no statistically significant difference in the Shannon Index (species richness/evenness) in any of the group comparisons.

**Figure 2:**
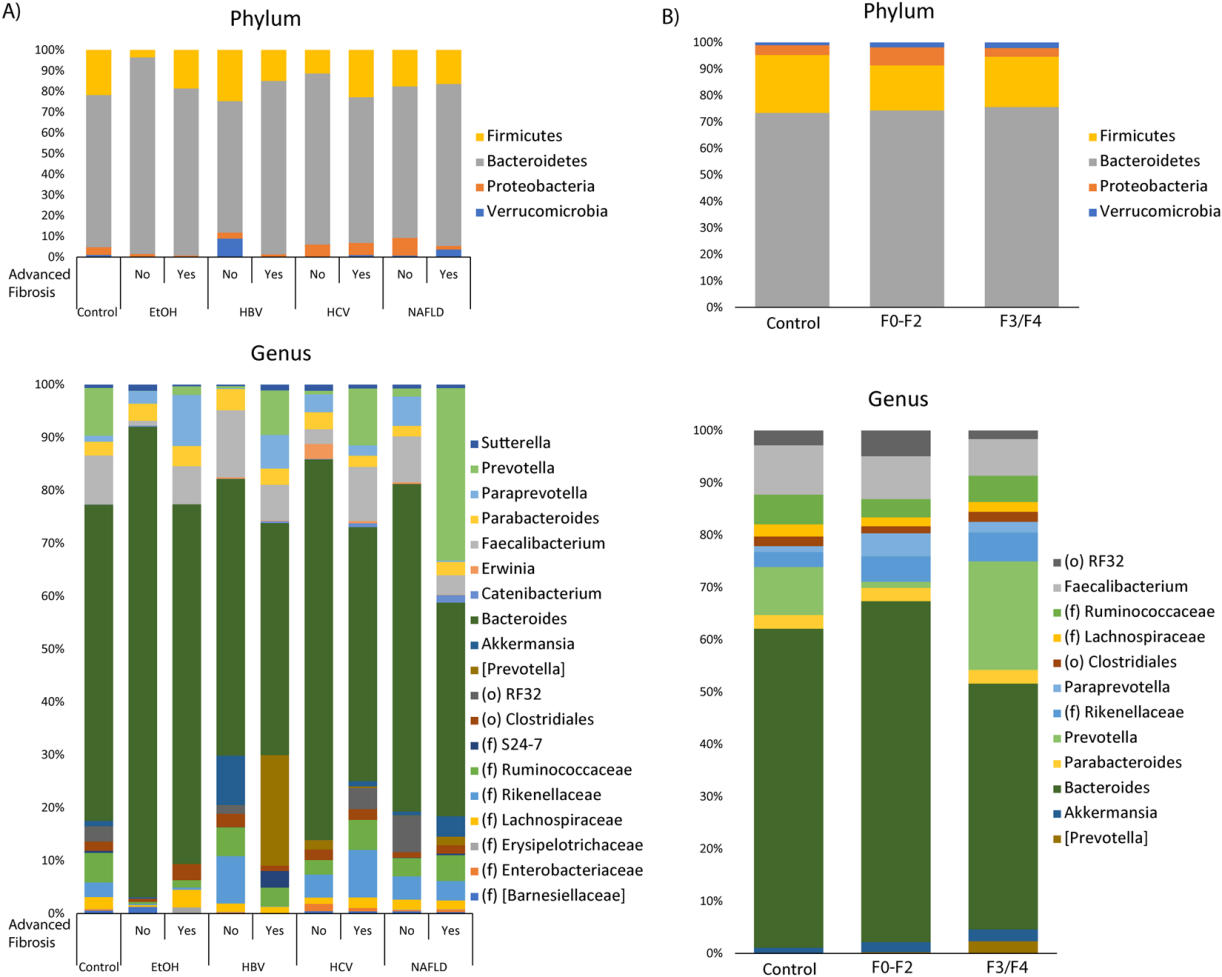
Taxonomic Profiles Categorized by Etiology of Chronic Liver Disease and Fibrosis Stage. (**A**) Taxonomic profiles at the phylum and genus levels, divided by etiology of chronic liver disease and fibrosis stage. (**B**) Taxonomic profiles by phylum and genus of patients with advanced fibrosis (F3/F4), liver patients with minimal/no fibrosis (F0-F2), and healthy controls. EtOH: Alcohol, HBV: Hepatitis B virus, HCV: Hepatitis C virus, NAFLD: nonalcoholic fatty liver disease.

The average taxonomic composition of chronic liver disease patients divided by etiology is summarized in Fig. 2A on a phylum and genus level. The composite taxonomic summary of all patients with advanced fibrosis, minimal or no fibrosis, or healthy controls is shown in Fig. 2B. Patients with alcoholic liver disease with F0-F2 fibrosis had a higher relative abundance of Bacteroidetes than any other group. Examining all patients with advanced fibrosis, there was a statistically higher abundance of *Prevotella* as compared to either healthy control or patients with F0-F2 disease as determined by differential abundance analysis adjusting for covariates.

**Figure 3:**
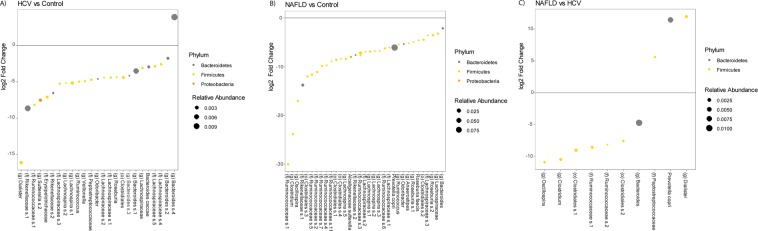
Microbial Communities Differ by Etiology of Chronic Liver Disease. DESeq2 differential abundance analysis comparing (**A**) HCV patients to control, (**B**) NAFLD patients to control, and (**C**) NALFD patients to HCV patients controlling for fibrosis. HCV: Hepatitis C virus, NAFLD: nonalcoholic fatty liver disease.

Differential abundance analysis adjusting for covariates was also performed to compare patients with different etiology of liver disease to healthy controls (Fig. 3). Because there were only 8 patients with alcoholic liver disease or HVC infection, the analysis only focused on patients with chronic HCV infection and NAFLD adjusting for fibrosis and the other covariates listed above. Patients with HCV disease as compared to controls differed significantly across 25 different OTUs (a taxonomic unit roughly corresponding to species). An undefined species belonging to the family Rikenellaceae, two undefined species in the genus *Bacteroides*, and an undefined species in the genus *Dialister* made up the OTUs with the largest relative abundance (Fig. 3A). NAFLD patients had 34 separate OTUs that were differentially abundant from healthy controls (Fig. 3B). The species with the highest relative abundance included *Prevotella copri*, an undefined species in the family Ruminococcaceae and an undefined species in the family Rikenellaceae. All 3 of these species were underrepresented in patients with NAFLD. Comparing NAFLD to HCV patients, there were 10 OTUs that were differentially abundant between the two groups. *Prevotella copri*, an undefined species belonging to the genus *Bacteroides*, and an undefined species of the order Clostridiales made three most abundant OTUs. *Prevotella copri* was higher in patients with NAFLD adjusting for fibrosis stage, while the other two OTUs were higher in patients with HCV (Fig. 3C).

**Figure 4:**
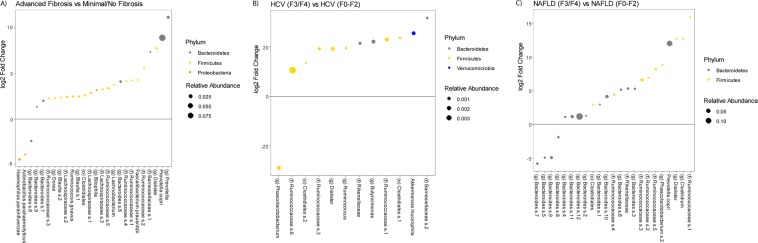
Microbial Communities Differ by Fibrosis Stage. DESeq2 differential abundance analysis comparing (**A**) advanced fibrosis patients to minimal/no fibrosis patients, (**B**) HCV patients with advanced fibrosis (F3/F4) to HCV patients without advanced fibrosis (F0-F2), and (**C**) NAFLD patients with advanced fibrosis to NAFLD patients without advanced fibrosis. HCV: Hepatitis C virus, NAFLD: nonalcoholic fatty liver disease.

Between patients with advanced fibrosis vs. minimal or no fibrosis, 26 OTUs were differentially abundant. The two most highly abundant differential OTUs were *Prevotella copri* and an undefined species belonging to the genus *Bacteroides*, both of which were elevated in patients with advanced fibrosis (Fig. 4A). Examining differences between fibrosis stage within patients with HCV and with NAFLD, there were 12 OTUs and 23 OTUs that were differentially abundant, respectively. While *Prevotella copri* did have a higher relative abundance in HCV patients with advanced fibrosis, it did not reach statistical significance. Instead, two undefined species in the family Ruminococcaceae and *Akkermansia muciniphila* were the three differential OTUs with the highest abundance; all three were elevated in patients with HCV with advanced fibrosis (Fig. 4B). In NAFLD patients, *Prevotella copri* was the predominant species and it was elevated in patients with advanced fibrosis (Fig. 4C).

### Predicted metagenomic profile differs by fibrosis

**Figure 5:**
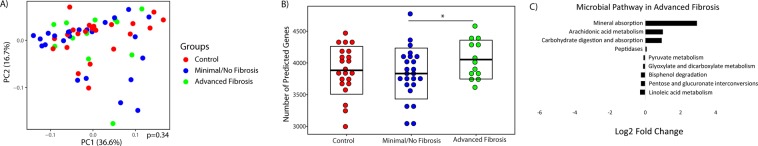
Predicted Metagenomic Differences by Fibrosis Stage. (**A**) Principal coordinates analysis plot of predicted metagenomic profiles between samples by fibrosis stage. (**B**) Number of predicted genes present per sample by fibrosis stage. Solid bar represents the mean and the box represents 1 standard deviation. *p = 0.09. (**C**) Differential abundance analysis (q < 0.05) of predicted metagenes for advanced fibrosis categorized by KEGG pathways predicted by PICRUSt from 16S rRNA data.

Metagenomic profiles were predicted for each sample from 16S rRNA compositional data using PICRUSt. The predicted metagenomic profile that differed between patients with advanced fibrosis as compared to those patients with minimal or no fibrosis is summarized in Fig. 5. The average weighted Nearest Sequenced Taxon Index (NTSI) per sample was 0.08. Low scores indicate availability of closely related reference genomes and thus a higher quality of predictions^26^. While there was no overall large difference of the predicted metagenome between samples by fibrosis stage as represented by the principal coordinates analysis in Fig. 5A (p = 0.34), patients with advanced fibrosis did have a trend to have more bacterial genes present per sample (Fig. 5B, p = 0.09). From 16S rRNA compositional data, DESeq2 analysis of PICRUSt predicted metagenes showed 168 metagenes that were statistically differentially expressed in patients with advanced fibrosis as compared to those with minimal or no fibrosis. Categorizing these metagenes into functional categories showed 9 pathways that are different between the two groups. The pathways that were most different between the two groups were those involved in mineral absorption, arachidonic acid metabolism, carbohydrate digestion and absorption, and linoleic acid metabolism (Fig. 5C).

### A microbial signature predicts advanced fibrosis

**Figure 6:**
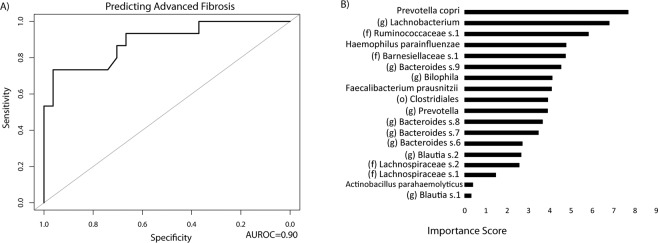
A Distinct Microbial Signature Can Accurately Identify Patients with Advanced Fibrosis. (**A**) Receiver operating characteristic curve of the random forests classifier for identifying patients with advanced fibrosis. (**B**) Importance scores for features included in the random forests classifier for predicting advanced fibrosis.

Using the 26 OTUs that were differentially abundant between patients with advanced fibrosis and patients with minimal or no fibrosis, a random forest classifier was created with high accuracy for predicting advanced fibrosis. The area under the receiver operating characteristic curve (AUROC) was 0.90 in 10-fold cross-validation (Fig. 6A). The contribution of each OTU to the classifier was expressed as variable importance score, which measures the decreased accuracy of the classifier if that feature was removed (Fig. 6B). The species with the greatest variable importance score was *Prevotella copri* followed by two undefined OTUs belonging to the genus *Lachnobacterium* and family Ruminococccaceae.

### A separate cohort validates the finding that a distinct microbial signature predicts advanced fibrosis

**Figure 7:**
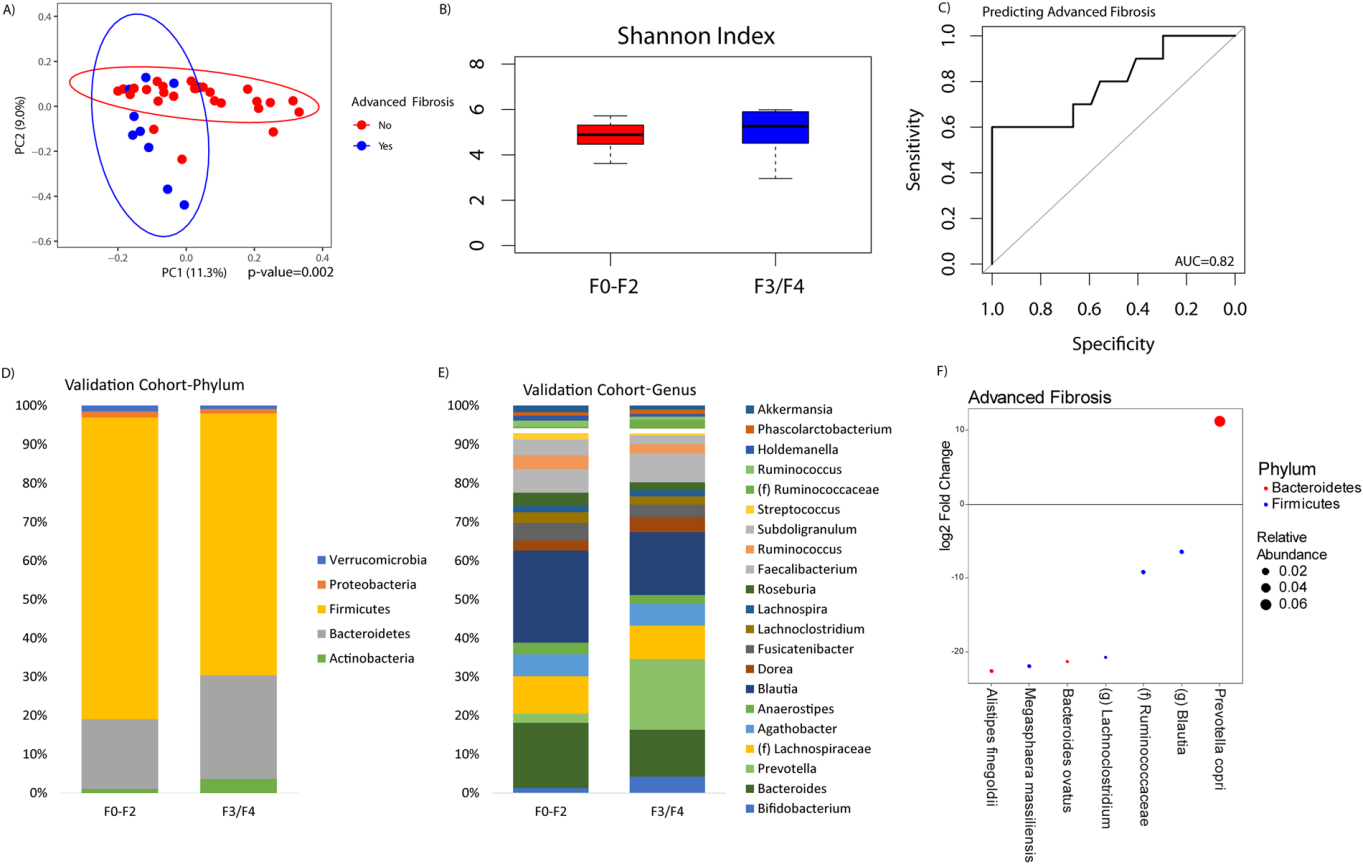
Validation of an advanced fibrosis microbial signature in a prospective study of NAFLD patients. (**A**) Principal coordinate analysis plot of beta diversity between patients with minimal to no fibrosis (F0-F2) versus patients with advanced fibrosis (F3/F4) within the validation cohort. (**B**) Shannon index of the validation cohort between patients with F0-F2 fibrosis and F3/F4 fibrosis. (**C**) Validation of the random forests classifier as depicted by a receiver operating characteristic curve. (**D**,**E**) Taxonomic profiles by phylum and genus of patients with minimal/no fibrosis (F0-F2) and patients with minimal/no fibrosis (F0-F2) within the validation cohort. (**F**) DESeq2 differential abundance analysis comparing advanced fibrosis patients to minimal/no fibrosis patients in the validation cohort.

In the validation cohort, there was no statistical difference between patients with advanced fibrosis as compared to minimal or no fibrosis in regards to age, gender, comorbidities, race, or dietary patterns (Table 2). Similar to the original cohort, a distinct microbial profile exists for patients with advanced fibrosis as compared to those with minimal or no fibrosis (Fig. 7). In univariate analysis of beta diversity, only age and advanced fibrosis had a p-value < 0.1. Therefore, these two variables were used for multivariate analysis. Adjusting for age, the microbial profile of patients with advanced fibrosis differed significantly as compared to those with minimal or no fibrosis as demonstrated in the principal coordinate analysis plot (p = 0.002). There was no statistical difference in Shannon index between patients with advanced fibrosis or those with minimal to no fibrosis in the validation cohort.

**Table 2:** Validation Cohort Characteristics.

Validation Cohort	F0-F2 (n = 27)	F3/F4 (n = 10)	p-value
Age (yr) (SD)	55.9 (11.0)	60.1 (6.7)	0.27
Male (%) (n = 29)	77.8% (n = 21)	80.0% (n = 8)	0.63
Charlson Comorbidity Index (SD)	2.7 (1.3)	2.9 (0.9)	0.99
Race/Ethnicity			
Caucasian (%) (n = 11)	29.6% (n = 8)	30.0% (n = 3)	0.23
African American (%) (n = 12)	40.7% (n = 11)	0% (n = 1)	
Hispanic (%) (n = 12)	25.9% (n = 7)	50.0% (n = 5)	
Asian (%) (n = 0)	0% (n = 0)	0% (n = 0)	
Other/Unknown (%) (n = 2)	3.7% (n = 1)	10.0% (n = 1)	
Dietary Data (intake per day)			
Alcohol (g)	1.81 (2.92)	1.59 (1.73)	0.82
Protein (g)	83.2 (42.4)	90.2 (44.5)	0.66
Total fat (g)	79.7 (50.9)	92.7 (63.1)	0.52
Total saturated fatty acids (g)	25.8 (16.0)	37.1 (33.5)	0.17
Total monounsaturated fatty acids (g)	29.4 (18.6)	31.4 (18.3)	0.78
Total polyunsaturated fatty acids (g)	17.4 (13.6)	16.0 (8.3)	0.78
Cholesterol (mg)	295.6 (197.0)	388.5 (221.0)	0.23
Carbohydrate (g)	244.9 (169.2)	247.9 (142.3)	0.96
Total sugars (g)	118.5 (97.0)	129.6 (88.9)	0.75
Dietary fiber (g)	21.6 (14.1)	20.8 (10.4)	0.87

SD: standard deviation.

The average taxonomic composition by fibrosis category is summarized in Fig. 7D,E, highlighting increased *Prevotella* in the advanced fibrosis group. Differential abundance testing demonstrated that 7 OTUs differed between patients with advanced fibrosis vs. minimal or no fibrosis. Of these, *Prevotella copri* was the most abundant and it was the only one that was enriched in those with advanced fibrosis. Applying the same random forest classifier trained on the initial cohort, microbiome composition had an AUROC of 0.82 for differentiating advanced vs. minimal or no fibrosis based on 10-fold cross-validation (Fig. 7C).

## Discussion

This study yielded several important findings. In patients with chronic liver disease, we show that those with advanced stages of fibrosis have a distinct microbiome signature compared to those with lesser stages of fibrosis. This held true regardless of etiology of the liver disease and after adjusting for other covariates. These differences are characterized by an increase in the genus *Prevotella* and a decrease in *Bacteroides*. Furthermore, by using these microbial differences, a highly accurate model based on stool analysis can be created to identify those with advanced fibrosis.

We also show that microbial signatures differ across different etiologies of chronic liver disease. Similar to prior published works^28,29^, chronic HCV infection is associated with a decrease in the order Clostridiales and family Ruminococcaceae in patients with advanced fibrosis. This study also builds on prior data from NAFLD patients. Within our cohort the most abundant species that were significantly different between healthy controls and NAFLD patients while adjusting for the level of fibrosis were *Prevotella copri*, an undefined species in the family Ruminococcaceae, and an undefined species in the family Rikenellaceae. This is similar to other prior works showing a reduction of *Ruminococcus* and *Prevotella* in non-cirrhotic NAFLD patients^18,30^. *Prevotella’s* reduction in non-cirrhotic NAFLD patients as compared to healthy controls is likely related to diet. Diets that are high in fats and animal protein as compared to diets that are rich in fiber have been shown to increase *Bacteroides* and decrease *Prevotella*^31,32^. This finding is therefore in line with previous works that have linked the gut microbiome to diet and non-cirrhotic fatty liver disease.

Though the idea of using stool as a novel biomarker for advanced fibrosis was recently explored and validated, it was only done in patients with NAFLD and did not include other etiologies^18^. In our cohort of racially diverse patients with varying etiologies of chronic liver disease, we show that the idea of using stool analysis to identify patients with advanced fibrosis is not only feasible but potentially highly accurate. While several other non-invasive methods are currently available for the diagnosis of advanced fibrosis including magnetic resonance elastography (MRE), transient elastography, and lab-based models, these modalities can have reduced accuracy in patients with diabetes or severe obesity^33^. Therefore, we propose that stool analysis can be a potentially accurate method when other modalities are limited. Combination of stool testing with other non-invasive tests including Fib-4 and NAFLD fibrosis scoring may also prove to be an important clinical tool to identify those patients who are more likely to progress to advanced fibrosis or cirrhosis.

In our model, we show that *Prevotella copri* was the predominant species predictive of advanced fibrosis. This was also true in our validation cohort as well. While *Prevotella copri* is still present in normal healthy controls, it is significantly higher in patients with advanced fibrosis, a trend that is consistent across all etiologies of chronic liver disease. This is similar to Qin *et al*. who showed that *Prevotella* was enriched in patients with cirrhosis as compared to healthy controls^16^. *Prevotella copri* is of great interest as it has been extensively studied in other inflammatory diseases^34^. It encodes a unique superoxide reductase which may provide resistance to or even the use of host-derived reactive oxygen species produced during inflammation^35^. Mice colonized with *P. copri* have increased inflammation in a colitis model induced by dextran sulfate sodium^36^. *In vitro* models have shown that *P. copri* can stimulate IL-6, IL-23, and IL-17, all cytokines associated with pro-inflammatory Th17 responses^37^. This has led many to believe that *P. copri* is a potential driver of inflammation and can even induce such inflammatory diseases as rheumatoid arthritis^34^. In a recent publication, *Prevotella copri* was also seen as the main bacteria associated with advanced fibrosis in NAFLD pediatric patients^38^. Our analysis also shows a distinct bacterial metagenomic profile for patients with advanced fibrosis. In our analysis, we show that the pathways that were most different between patients with advanced fibrosis compared to those without were related to mineral absorption, arachidonic acid metabolism, carbohydrate digestion and absorption, and linoleic acid metabolism. In mouse models of liver steatosis, linoleic acid was shown to be protective against inflammation by affecting PPAR-α and NF-κβ signaling^39^. The observed associations of *P. copri* and these functional pathways with advanced fibrosis provide preliminary evidence that the gut microbiome may contribute to the progression of liver fibrosis. Therefore, it can be both a useful non-invasive biomarker as well as a potential target for future interventions.

We acknowledge that there were several limitations to this study. For example, we relied on FibroScan rather than liver histology to make the diagnosis of hepatic fibrosis. With the wide adoption of non-invasive testing for fibrosis, the use of liver biopsy is becoming less frequent. However, FibroScan is becoming a more widely accepted and accurate method for detecting the presence of hepatic fibrosis^40^. While other papers have mentioned that obesity might be a limitation of FibroScan, our facility and technicians had access to and were familiar with the XL probe, which has been proven to have improved diagnostics in obese patients^40^. Another limitation is that this is a single center VA study and so the generalizability of this study to other settings is still uncertain. While the multivariate analyses did not control for all factors that could affect the microbiome, including diet and medications, the corroboration of our findings in a separate validation cohort that accounted for diet strengthens the findings of our study. Furthermore, while we did attempt to represent a wide array of chronic liver disease, the majority of our patients had chronic HCV or NAFLD. A complete representation of all etiologies of chronic liver disease was unable to be accomplished due to the rarity of less common etiologies including autoimmune disease, Wilson’s disease, hemochromatosis, PSC, PBC, and alpha-1 antitrypsin deficiency. Therefore, future studies will be needed in order to confirm that these findings apply to other chronic liver disease etiologies. Because this study is cross-sectional, it is unable to establish causality between the gut microbiome and hepatic fibrosis. Planned future studies will include the use of fecal metabolomics to examine the differential pattern of microbial derived metabolites in patients with advanced fibrosis and the use of animal models with microbial transplant or single bacteria gavage to understand the causal relationship between the gut microbiome and hepatic fibrosis.

In conclusion, there is a distinct microbial signature for patients with advanced fibrosis independent of liver disease etiology and other comorbidities. These results suggest that microbial profiles can be used as a non-invasive marker for advanced fibrosis and support the hypothesis that microbes and their metabolites contribute to hepatic fibrosis. Future studies should focus on the mechanism by which these microbial differences may contribute to the progression of fibrosis and if the models presented here are valid in other clinical subgroups.
